# PELP1 Suppression Inhibits Colorectal Cancer through c-Src Downregulation

**DOI:** 10.1155/2014/193523

**Published:** 2014-05-22

**Authors:** Zhifeng Ning, Youzhi Zhang, Hanwei Chen, Jiliang Wu, Tieshan Song, Qian Wu, Fuxing Liu

**Affiliations:** ^1^Hubei Province Key Laboratory on Cardiovascular, Cerebrovascular, and Metabolic Disorders, Hubei University of Science and Technology, Xianning 437100, China; ^2^The Basic Medical School, Hubei University of Science and Technology, Xianning 437100, China; ^3^School of Pharmacy, Hubei University of Science and Technology, Xianning 437100, China; ^4^Wuhan Blood Center, Wuhan 430030, China

## Abstract

Proline-, glutamic acid-, and leucine-rich protein 1 (PELP1), a coregulator of estrogen receptors alpha and beta, is a potential protooncogene implicated in several human cancers, including sexual hormone-responsive or sexual hormone-nonresponsive cancers. However, the functions of PELP1 in colorectal cancer remain unclear. In this study, western blot and bioinformatics revealed that PELP1 expression was higher in several colorectal cancer cell lines than in immortalized normal colorectal epithelium. PELP1 silencing by short hairpin RNA promoted the senescence and inhibited the proliferation, colony formation, migration, invasion, and xenograft tumor formation of the CRC cell line HT-29. Moreover, PELP1 silencing was accompanied by c-Src downregulation. c-Src upregulation partly alleviated the damage in HT-29 malignant behavior induced by PELP1 RNA interference. In conclusion, PELP1 exhibits an oncogenic function in colorectal cancer through c-Src upregulation.

## 1. Introduction

Colorectal cancer (CRC) is the second leading cause of cancer-related death in the developed countries [1, 2]. In China, the morbidity and mortality rates of CRC have rapidly increased over the past two decades; these rates are affected by many factors, including age, population, westernization lifestyle, and industrial pollution. Early-stage CRC lacks specific symptoms; thus, this disease is usually diagnosed at a relatively late stage. The five-year survival rate of early-stage CRC is 85% after surgical resection, whereas that of stage III CRC with lymph node metastasis is <50% after surgery [3]. Cancer results from the activation of numerous protooncogenes and the inhibition of tumor suppressors. CRC is characterized by the mutation of tumor suppressor genes, protooncogenes, DNA repair genes, growth factors and their receptor genes, apoptosis-related genes, and cell cycle checkpoint genes [4]. These diverse genes constitute a complicated regulated network implicated in CRC oncogenesis. However, interference with a single gene is not enough to cure cancer. Therefore, the master regulators of downstream effector genes involved in carcinogenesis must be urgently identified [5].

The specific functions of estrogen signaling in CRC remain unclear. Many studies implicated the dysregulation of estrogen receptors (ERs) in CRC [6–9]. However, the specific functions of ER*α* and ER*β* in the cancerogenesis of colorectal epithelium remain controversial. Some researches [6, 8] implied that ER*β* is the predominant form expressed in the normal colorectal epithelium and reduced in CRC. Thus, ER*β* exerts protective effects against CRC; this finding is contradictory to the report that the ER*β* protein is significantly upregulated in colorectal epithelial cells of carcinomas [10]. ER*α* is reportedly rarely expressed in normal colorectal epithelium and lacks functions in CRC carcinogenesis. However, several studies reported that the ER*α* gene is frequently hypermethylated in CRC [11, 12], suggesting that ER*α* hypermethylation can predict CRC progression. ER*α* upregulation in ER*α*-negative CRC cell lines can suppress cell growth [13]. The reversal of a hypermethylated inactive ER*α* can inhibit CRC in vitro and in vivo [14]. The estrogen-ER signaling pathway is evidently involved in CRC development. Therefore, comprehensive research must be conducted to elucidate the functions of ER in CRC.

Proline-, glutamic acid-, and leucine-rich protein 1 (PELP1) is a modulator of nongenomic actions of estrogen receptors (NMAR) and a coregulator of ER; PELP1 has been implicated in many physiological [15–17] and pathological processes [18]. Research has demonstrated that PELP1 is a protooncogene in hormone-responsive cancers, such as breast [19, 20], ovarian [21], endometrial [22, 23], and prostate cancers [24]. Moreover, some researchers determined the functions of PELP1 in hormone-nonresponsive cancers, such as brain tumor [25], lung cancer [26], and colorectal cancer [27, 28].

PELP1 is upregulated in CRC tissues [27, 28]. However, the exact function of PELP1 in CRC remains unknown. In the present study, we investigated the functions of PELP1 in several CRC cell lines. Bioinformatics and western blot showed that PELP1 expression was higher in CRC cell lines than in normal colorectal epithelium. PELP1 silencing by short hairpin RNA (shRNA) promoted the senescence and inhibited the proliferation, colony formation, migration, invasion, and xenograft tumor formation of the CRC cell line HT-29. Moreover, PELP1 silencing was accompanied by c-Src downregulation. c-Src upregulation partly recovered the oncogenic function of PELP1. These results demonstrated that PELP1 suppression can inhibit CRC in vitro and in vivo through c-Src downregulation.

## 2. Materials and Methods

### 2.1. Cell Line

The CRC cell lines COLO205, HT-29, SW-620, HCC-2998, and HCT-15 and normal colon epithelium cell line FHC were purchased from American Type Culture Collection (Manassas, VA). The five CRC cell lines were cultured in RPMI1640 medium supplemented with 10% fetal bovine serum, 100 U/mL penicillin, and 100 *μ*g/mL streptomycin. FHC was cultured in DMEM/F12 medium with the same supplement as the CRC cell lines. All cell lines were maintained at 37°C in a humidified atmosphere of 5% CO_2_ in an open-air incubator.

### 2.2. Bioinformatics

We performed a bioinformatics analysis on Oncomine (https://www.oncomine.org/resource/login.html) to analyze PELP1 expression in the CRC cell lines. We used a personal account to enter the Oncomine website. We input PELP1 on the search box to begin the search. After entering the page for PELP1 expression survey in almost all human cancer cell lines or tissues, we focused on CRC and obtained PELP1 expression data from the Lee et al. [29], Shankavaram et al. [30], and Garnett [31] cell line datasets. After data standardization, we presented PELP1 expression data in the five CRC cell lines as mean ± standard deviation (SD).

### 2.3. shRNA Interference of PELP1

HT-29 cells were seeded in 24-well plates (Corning) until they reached 50% to 60% confluence prior to transfection. Then, stable transfection was performed. Three shRNA sequences targeting the PELP1 gene were designed and synthesized by Shanghai Jima Pharmaceutical Technology, China. The three shRNA sequences of PELP1 and the negative control sequence are listed in Table 1. Lipofectamine LTX and Plus Reagent (Invitrogen) were used to transfect shRNA into HT-29 cells according to the manufacturer's protocol. The medium containing transfection reagents was replaced with RPMI1640 medium supplemented with 10% FBS at 18 h after the transfection. The cells were collected at 48 h after the transfection, processed in the following experiments, and then prepared for protein extraction. The silence efficiency of PELP1 was tested by western blot.

### 2.4. Western Blot

Total protein was extracted from HT-29-shRNA or HT-29-control cells using RIPA lysate buffer as described in our previous research [32]. After quantification by a bicinchoninic acid protein assay kit (Beyotime Biotechnology, China), equal amounts of proteins (20 *μ*g to 25 *μ*g) were separated by 12% sodium dodecyl sulfate-polyacrylamide gel electrophoresis (PAGE) and processed for immunoblotting with a rabbit multiclonal antibody for PELP1 (diluted at 1 : 100, Santa Cruz) and c-Src (diluted at 1 : 500, Santa Cruz). A mouse polyclonal anti-*β*-actin antibody (diluted at 1 : 2000, Boster Biological Engineering, China) was used as an internal control. All protein bands were scanned using ChemiImager 5500 V2.03 software, and the integrated density values were calculated by a computerized image analysis system (Fluor Chen 2.0) and normalized with that of *β*-actin.

### 2.5. MTT

Cell viability and proliferation activity were determined with the MTT assay. HT-29-shRNA or HT-29-control cells were seeded in 96-well plates (Costar) at a density of 5,000 cells per well in complete medium (RPMI1640 supplemented with 10% FBS, 1% p/s) and then incubated for 12 h under standard conditions (37°C and 5% CO_2_). The total volume in each well was 200 *μ*L. From the next day to the seventh day, 20 *μ*L of MTT (5 mg/mL) was added into each well. After additional incubation for 4 h, the solution in each well was replaced with dimethyl sulfoxide (Sigma, USA) to solubilize formazan, the metabolic product of MTT. The plates were kept on a shaking mixer for 10 min to guarantee complete solubilization of formazan, and the optical density was recorded at 490 nm using a microplate luminometer. Results were expressed as means ± SD, and a growth curve was constructed. Data were analyzed by one-way ANOVA with the post hoc Tukey test applied for paired comparisons.

### 2.6. Plate Colony Forming Assay

Colony forming ability was examined by the plate colony formation assay. HT-29-shRNA or HT-29-control cells were seeded into six-centimeter plates (Costar) at a density of 3,000 cells per plate in complete medium and then incubated for approximately 2 weeks under standard conditions (37°C and 5% CO_2_). The cells were washed twice with phosphate-buffered saline (PBS) and then fixed with methanol for 15 min. After staining with 0.1% crystal violet for 20 min, the number of positive colonies with diameters exceeding 50 *μ*m was counted under a light microscope with 100× magnification. The colony forming rate was calculated by dividing the number of positive colonies by the total number of cells seeded.

### 2.7. Transwell Small Chamber Invasion and Migration Assay

For the invasion assay, Transwell small chambers with 8 *μ*m pore filters were coated with 12 *μ*L of ice-cold Matrigel (7.5 mg/mL protein). In total, 50,000 HT-29-shRNA or HT-29-control cells were added to the upper chamber of these Matrigel chambers in 200 *μ*L of serum-free RPMI 1640 medium. Then, the cells were placed in 24-well plates in 500 *μ*L of RPMI 1640 medium containing 10% FBS. After 22 h of incubation, the cells were fixed with methanol and then stained with 0.1% crystal violet. Cotton tips were used to remove the cells that remained in the Matrigel or attached to the upper side of the filter. The number of cells on the lower side of the filter was counted under a light microscope.

The methods used for the migration assay were almost the same as for the invasion assay described above, except that no Matrigel was used to coat the well and the incubation time was 16 h.

### 2.8. *β*-Galactosidase (*β*-Gal) Staining

The cells were washed in PBS, fixed for 3 min to 5 min (room temperature) in 2% formaldehyde/0.2% glutaraldehyde (or 3% formaldehyde), washed again, and then incubated at 37°C (no CO_2_) with a fresh senescence-associated *β*-Gal stain solution containing 1 mg of 5-bromo-4-chloro-3-indolyl *β*-D-galactoside per mL (stock = 20 mg of dimethylformamide per mL)/40 mM citric acid/sodium phosphate, pH 6.0/5 mM potassium ferrocyanide/5 mM potassium ferricyanide/150 mM NaCl/2 mM MgCl_2_. Staining was evident within 2 h to 4 h and peaked within 12 h to 16 h. To detect lysosomal, *β*-Gal, the citric acid/sodium phosphate was pH 4.0.

### 2.9. Nude Mice Subcutaneous Xenograft Assay

Male BALB/c (nu/nu) mice aged 6 to 8 weeks were obtained from the Experimental Animal Center of Guangdong Province. The mice were housed and maintained in laminar flow cabinets under specific pathogen-free conditions according to the regulations and standards approved by the Animal Care and Ethics Committee of Shantou University Medical College.

To establish s.c. tumors, 1.5 × 10^6^ HT-29-shRNA or HT-29-control cells were resuspended in 200 *μ*L of RPMI1640 serum-free medium and injected via an 18-gauge needle into the s.c. space of both flanks of the mice. Tumor progression was documented once weekly by measurements using calipers, and tumor volumes were calculated by the following formula: length × width × height × 0.52 (in mm). The mice were given ethane anesthesia and then euthanized by cervical dislocation.

### 2.10. Stable Transfection of c-Src

The human c-Src coding region gene with a 376 bp sequence was amplified from homogenized HT-29 by RT-PCR. The sequences of the PCR primers used for c-Src in this study were as follows: sense, 5′-TGTTCGGAGGCTTCAACTCC-3′ and antisense, 5′-CAGTAGGCACCTTTCGTGGT-3′. The PCR products were cloned into a TA expression vector (Invitrogen, Carlsbad, CA, USA), and the sequence of the c-Src coding region was confirmed by sequencing. The resulting plasmids (pc-Src) were propagated in* Escherichia coli* and then purified through cesium chloride gradient. For gene transfection, HT-29-shRNA or HT-29-control cells were seeded in six-well plates at a concentration of 5 × 10^5^ cells per well. HT-29-shRNA or HT-29-control cells approaching 80% to 90% confluence were transfected with 4 mg pc-Src with 10 mL of Lipofectamine 2000 (Invitrogen) following the manufacturer's protocol. The cells transfected with empty plasmid pcDNA3.1 (mock) were used as negative controls.

### 2.11. Quantitative RT-PCR

The cells or tissues were harvested with Trizol Reagent (Invitrogen, Carlsbad, CA, USA), and total RNA was isolated according to the manufacturer's instructions. cDNA synthesis was performed using the Superscript III RT-PCR kit (Invitrogen). Real-time PCR was carried out using a Cepheid SmartCycler II (Sunnyvale, CA, USA) with gene-specific real-time PCR primers. Results were normalized to GAPDH transcript levels, and the difference in fold expression was calculated using the ΔΔ CT method. The primers used for c-Src were as follows: sense, 5′-CTCTTCAGAGCCCTTGCTCA-3′ and antisense, 5′-ATTCACCCTCCCCCAAGGAA-3′. The length of the PCR products was 193 bp.

### 2.12. Statistics Analysis

Data from all quantitative assays were expressed as mean ± SD andwere analyzed using one-way ANOVA and independent-samples *t*-test. All statistical analyses were performed and visualized by GraphPad Prism 5.0. *P* < 0.05 was considered statistically significant.

## 3. Results

### 3.1. PELP1 Was Upregulated in CRC Cell Lines as Revealed by Western Blot and Bioinformatics

**Figure 1:**
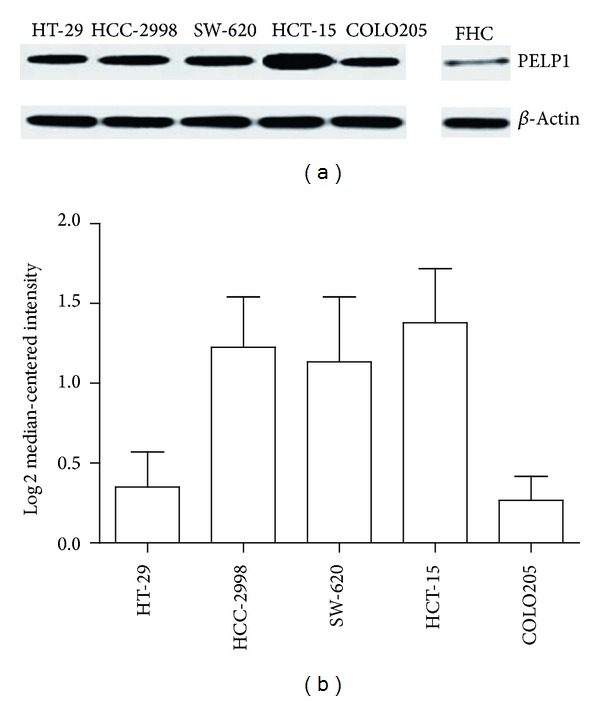
PELP1 expression was upregulated inCRC. (a) Western blot revealed that PELP1 protein expression was higher in the CRC cell lines HT-29, HCC-2998, SW-620, HCT-15, and COLO205 than in the normal colorectal epithelium FHC. (b) Informatics data suggested that PELP1 mRNA expression was increased in these five CRC cell lines.

We performed western blot to test the protein expression of PELP1 in the CRC lines COLO205, HT-29, SW-620, HCC-2998, and HCT-15 and in the normal cell line FHC. As shown in Figure 1(a), PELP1 protein expression was higher in the CRC cell lines than in the immortalized colorectal epithelium. The bioinformatics data (Figure 1(b)) obtained from the Oncomine database also showed that PELP1 was upregulated in the CRC cell lines. These results implied that PELP1 served oncogenic functions in CRC.

### 3.2. PELP1 Downregulation by shRNA Inhibited CRC In Vitro

**Figure 2:**
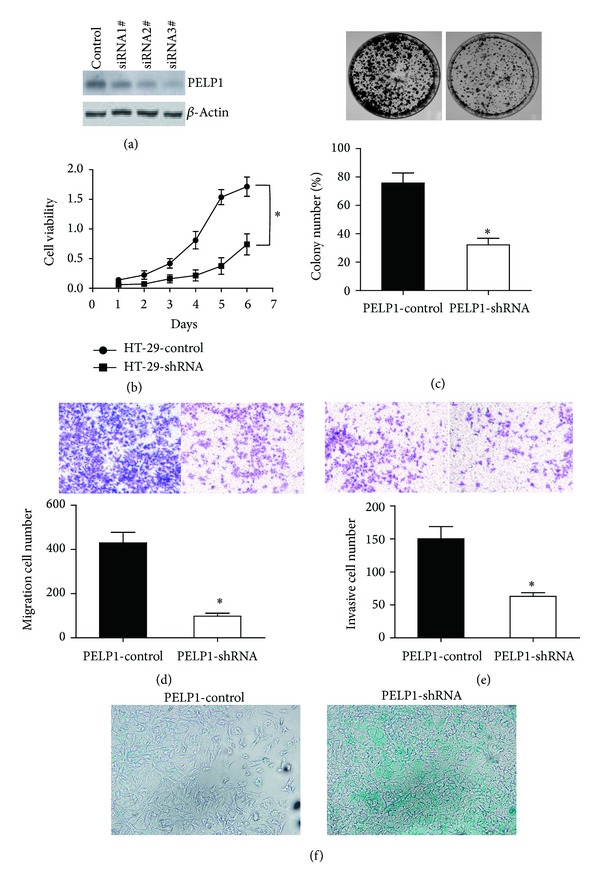
PELP1 downregulation inhibited the CRC cell line HT-29 in vitro. (a) After transfection with shRNA, PELP1 protein expression was decreased by 90%. shRNA #3 was selected for further investigations. (b) After PELP1 silencing, the cell viability of HT-29 was inhibited. (c) PELP1 silencing inhibited the colony formation ability of HT-29 by 57.5% (lower panel). A representative colony formation assay is shown (upper panel). (d) PELP1 silencing inhibited the migration ability of HT-29 by 69.3% (lower panel). A representative migration assay is shown (upper panel). (e) PELP1 silencing inhibited the invasion ability of HT-29 by 58% (lower panel). A representative invasive assay is shown (upper panel). (f) Senescence was induced by PELP1 silencing in HT-29. A representative *β*-Gal assay is shown.

To identify the oncogenic function of PELP1 in CRC, we utilized shRNA to silence PELP1 expression in HT-29. After transfection shRNA #3 of PELP1 into HT-29, PELP1 expression decreased by 90% (Figure 2(a)). PELP1 silencing reduced the proliferation, colony formation (by 57.5%), migration (by 69.3%), and invasion (by 58%) abilities of HT-29 (Figures 2(b)–2(e)). *β*-Gal senescence assay demonstrated that PELP1 silencing promoted the senescence of HT-29 (Figure 2(f)). These results suggested that PELP1 downregulation inhibited the malignant behavior of CRC in vitro.

### 3.3. PELP1 Downregulation by shRNA Inhibited Xenograft Formation Ability of CRC in Nude Mice

**Figure 3:**
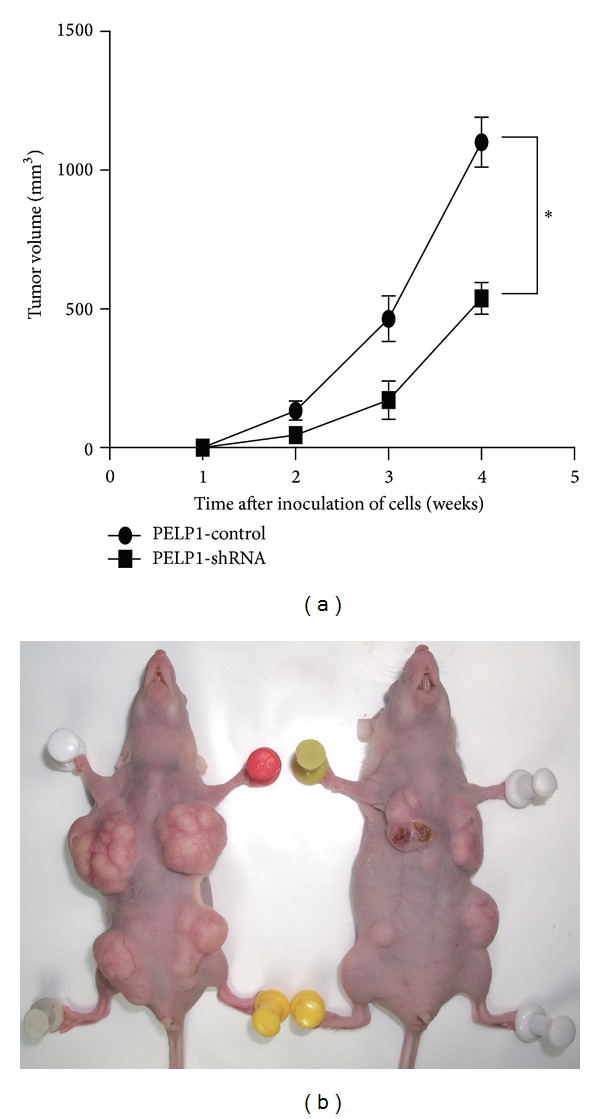
PELP1 downregulation inhibited the CRC cell line HT-29 in nude mice xenograft assay. PELP1 silencing inhibited CRC growth in nude mice (a). A representative nude mice xenograft assay is shown in (b).

Xenograft formation ability reflects the malignant characteristic of cancer cells. We performed a subcutaneous xenograft formation experiment on nude mice to assess whether or not PELP1 can influence the xenograft formation ability of HT-29. After stable transfection with PELP1-shRNA in HT-29, HT-29-control and HT-29-shRNA cells were inoculated subcutaneously in both flanks of the nude mice. Results showed that PELP1-shRNA reduced the xenograft formation ability of HT-29 in nude mice (Figure 3).

### 3.4. Oncogenic Function of PELP1 in CRC Was Mediated by c-Src Upregulation

**Figure 4:**
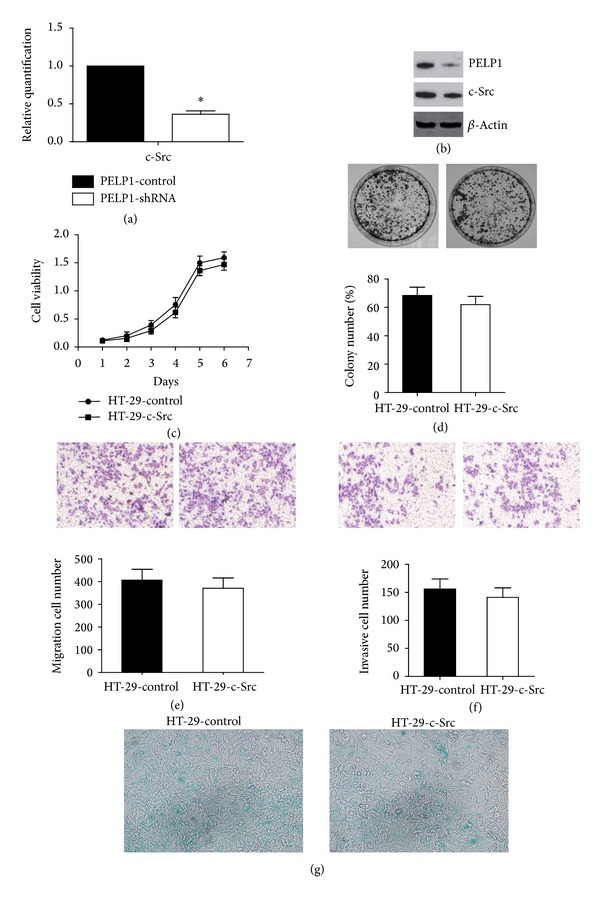
PELP1 silencing suppressed CRC through c-Src downregulation. (a) PELP1 silencing was accompanied by the downregulation of c-Src mRNA as determined by quantitative RT-PCR. (b) PELP1 silencing was accompanied by c-Src protein downregulation as determined by western blot. (c) Decreased cell viability induced by PELP1 silencing was recovered by c-Src upregulation in HT-29. (d) Decreased colony formation ability was recovered by c-Src upregulation in HT-29. (e) Decreased migration ability was recovered by c-Src upregulation in HT-29. (f) Decreased invasion ability was recovered by c-Src upregulation in HT-29. (g) Induced senescence by PELP1 silencing was inhibited after c-Src upregulation.

c*-*Src is a known protooncogene in colon cancer. Thus, we focused on the relationship between PELP1 and c-Src in CRC carcinogenesis. We tested c-Src expression by quantitative RT-PCR and western blot after PELP1 silencing by shRNA in HT-29 to explore the mechanism of PELP1 downregulation in suppressing CRC carcinogenesis. c-Src was reduced after PELP1 silencing at the mRNA (Figure 4(a)) and protein (Figure 4(b)) expression levels. To analyze whether or not c-Src participates in promoting the function of PELP1 in CRC, we upregulated the expression of c-Src after PELP1 silencing. As stated previously, PELP1 downregulation can inhibit the malignant behavior of CRC. Surprisingly, Src elevation can counteract the inhibition of CRC induced by PELP1 silencing (Figures 4(c)–4(g)). Therefore, the suppressing function of PELP1 downregulation in CRC carcinogenesis was mediated by c-Src inhibition.

## 4. Discussion

In the present study, the oncogenic function of the ER coregulator PELP1 or NMAR was identified in CRC, a common and highly invasive type of cancer. We primarily clarified the oncogenic mechanism of PELP1 in relation to c-Src, an important oncogene. Although CRC cannot be completely regarded as a hormone-responsive cancer, emerging evidence implied that steroid hormones such as estrogen and androgen participate in CRC pathogenesis. However, the functions of ER and AR in CRC are complex and controversial because many complicated signaling pathways are involved in estrogen and androgen signaling. ER*β* is usually regarded as a protective factor in CRC, and ER*α* is believed to have no participation in CRC [33]. Different from ERs, androgen receptors (ARs) serve no [34] suppressive [35] or promoting functions [36] in CRC. As a nuclear receptor coregulator, PELP1 can interact with ERs, ARs, glucocorticoid receptors, and progesterone receptors [37]. Therefore, we analyzed the functions of PELP1 in CRC to elucidate the complex signaling network involving ER and AR.

Two recent studies have evaluated the expression of PELP1 in CRC tissues by using immunohistochemistry [27, 28]. However, their findings were inconsistent. Tzelepi et al. [27] claimed that PELP1 expression decreases from normal colorectal epithelium to cancerous colorectal epithelium; by contrast, Grivas et al. [28] found that PELP1 protein expression in epithelial cells increases during colorectal tumorigenesis despite the fact that PELP1 overexpression in epithelial cells correlates with prolonged overall survival. Moreover, Grivas et al. [28] found that ER*β* expression in epithelial cells is upregulated during colorectal tumorigenesis in male patients. They also found that ER*β* expression correlates with increased risk of relapse, implying that ER*β* serves an oncogenic function in male CRC patients. The present experimental results seemed to be partly consistent with those of Grivas et al. That is, bioinformatics and western blot assay demonstrated that the mRNA and protein expression levels of PELP1 increased in the CRC cell lines COLO205, HT-29, SW-620, HCC-2998, and HCT-15. Moreover, we found that the malignant behavior of CRC was inhibited in vitro and in vivo after PELP1 silencing by shRNA. These results suggested that PELP1 served an oncogenic function in CRC.

A decade ago, Peyton Rous described a filterable agent (i.e., virus) that can induce solid tumor formation in birds. After approximately 50 years, the Rous* sarcoma virus* was identified from Rous' filterable agent. Extensive research into the molecular biology and genetics of Rous* sarcoma virus* identified v-Src as a viral oncogene responsible for malignant transformation. Then, Bishop and Varmus [38] demonstrated that v-Src has a cellular counterpart, the first identified protooncogene c-Src. c-Src is a nonreceptor tyrosine kinase that is abnormally expressed in many human cancers and is linked with malignant biological behavior related to proliferation, adhesion, migration, invasion, and metastasis [39]. The oncogenic function of c-Src in CRC has been explored in different studies. The dysregulation of c-Src contributed to the initiation and development of CRC. In the present study, we linked PELP1 with c-Src in CRC. Several scholars have identified the fact that an interaction exists between PELP1 and c-Src. For example, Chakravarty et al. [40] found that estrogen-mediated extranuclear signaling promotes cytoskeleton reorganization through the ER-Src-PELP1-phosphoinositide 3-kinase-ILK1 pathway in breast cancer. PELP1 silencing can significantly inhibit c-Src activation. Dimple et al. [41] suggested that PELP1 downregulation reduces the proliferation and tumorigenic potential of ovarian cancer cells and affects the magnitude of c-Src and protein kinase B (AKT) signaling in a nude mouse model. Rajhans et al. [42] found that the PELP1-mediated induction of aromatase requires functional Src and phosphatidylinositol-3-kinase pathways. Conversely, c-Src phosphorylates PELP1 at the C terminal (tyrosine 920) domain [43]. However, PELP1 can function independent of c-Src. Kayahara et al. [44] found that PELP1 functionally interacts with both NH2- and COOH-terminal glucocorticoid receptor domains to modulate transactivation; they also found through inhibitor and c-Src knockdown studies that the function of PELP1 is independent of c-Src activity. In the present study, we found that the oncogenic function of PELP1 was partially mediated by c-Src in CRC. PELP1 silencing by shRNA downregulated c-Src expression. In addition, c-Src upregulation partly recovered the oncogenic function of PELP1. Our research suggested that the oncogenic effect of PELP1 in CRC was partly mediated by c-Src.

## 5. Conclusions

PELP1, a nuclear receptor coregulator, exerts oncogenic action in CRC. PELP1 silencing by shRNA promoted the senescence and inhibited the proliferation, colony formation, migration, invasion, and xenograft tumor formation of CRC. Moreover, PELP1 silencing was accompanied by c-Src downregulation. c-Src upregulation partly recovered the oncogenic function of PELP1. This study is the first to identify the oncogenic function of PELP1 in CRC, a hormone-nonresponsive cancer. Therefore, PELP1 can be regarded as a therapeutic target in hormone-nonresponsive cancers.

## Figures and Tables

**Table 1:** Interference sequence for shRNA.

shRNA number	Sequence (3′-5′)
Negative control	GCAAGCTGACCCTGAAGTTGAGAACTTTGAAGTCCCAGTCGAACG
1	GGAGAAACAAAGGUUAUUUGAGAACTUUUAUUGGAAACAAAGAGG
2	CACAGUCUCACAUCGUUUAGAGAACTAUUUGCUACACUCUGACAC
3	CCAGGAGCTTGTTGAAGAAGAGAACTAAGAAGTTGTTCGAGGACC

## Abbreviations

PELP1: Proline-, glutamic acid-, and leucine-rich protein 1
c-Src: Cellular counterpart of Rous sarcoma virus
ER: Estrogen receptor
CRC: Colorectal cancer.
